# SACE and IL-2R as serum biomarkers for evaluation of multi-organ involvement and prognosis of sarcoidosis

**DOI:** 10.1186/s12931-023-02524-0

**Published:** 2023-09-07

**Authors:** Ying Zhou, Xianqiu Chen, Mengmeng Zhao, Elyse E. Lower, Robert P. Baughman

**Affiliations:** 1 Department of Pulmonary and Critical Care Medicine, Shanghai Pulmonary Hospital, Tongji University School of Medicine, 507 Zheng Min Road, Shanghai, 200433 China; 2 Department of Medicine, University of Cincinnati Medical Center, Cincinnati, OH USA

**Keywords:** Sarcoidosis, Serum angiotensin converting enzyme, Interleukin-2 receptor, Organ involvement, Prognosis

## Abstract

**Background:**

Serum biomarkers in the evaluation of organ involvement and prognostic monitoring of sarcoidosis have not been determined. The purpose of this study was to identify common biomarkers that could be used to assess organ involvement and monitor outcomes in sarcoidosis patients.

**Methods:**

From Mar 2013 to Sep 2021, patients with newly diagnosed pulmonary sarcoidosis were enrolled in this study in Shanghai Pulmonary Hospital. The information from medical records was retrospectively collected including diagnosis, organ involvement, laboratory tests and follow up data. Differences of continuous variables between groups were analyzed by unpaired Student’s t-test. Multivariate logistic regression model was performed to identify potential independent factors associated with multiple organ involvement.

**Results:**

A total of 832 patients were included in the study. There were 339 (40.7%) patients with single organ pulmonary involvement, while 493 (59.3%) patients had two to seven organs involved. Among the routine serum tests, only the serum angiotensin converting enzyme (SACE) level was an independent factor of multiple organ involvement. Compared to those patients without involvement, SACE levels were higher in patients with extra-thoracic lymph node, skin, or spleen involvement as well as abnormal calcium metabolism. Interleukin-2 receptor (IL-2R) levels were higher in patients with extra-thoracic lymph node, spleen involvement and abnormal calcium metabolism than in those without it. The mean levels of SACE and IL-2R showed upward trends paralleling the increase on number of organs involved. In follow up, SACE and IL-2R levels were both decreased in an improved patient group, while there was no obvious difference was noticed before and after treatment in patients with persistent disease.

**Conclusion:**

SACE and IL-2R were useful as serum biomarkers in the initial evaluation of organ involvement as well as monitoring prognosis in sarcoidosis.

## Introduction

Sarcoidosis is a systemic granulomatous disorder of unknown etiology that may affect almost every body organ. The most commonly involved organs remain the lung and mediastinal lymph nodes. The incidence of sarcoidosis varies due to different regions, races and demographics, and recent epidemiologic studies suggest that the annual incidence of sarcoidosis varies between 1 and 15 per 100,000 [1]. Clinical manifestations of sarcoidosis are various on the basis of the race, gender, as well as the extent and severity of organ mainly affected [2]. Because there is no specific test or algorithm for the diagnosis of sarcoidosis, its diagnosis depends on a compatible clinical and imaging feature, histologic evidence of non-necrotizing epithelial granuloma, and exclusion of alternative causes of granulomatous diseases [3]. Therefore, identifying biomarkers is warranted to assist in disease diagnosis and disease activity surveillance, as well as improve prognostic outcomes for patients with sarcoidosis.

Because of simple sample collection, minimal invasiveness, and low cost, the serum markers can be of great value in disease prediction and prognosis outcome assessment. Many biomarkers and their correlation with sarcoidosis have been reported in previous studies [4]. Serum angiotensin converting enzyme (SACE) is abnormally upregulated in some patients with sarcoidosis, and it could assist in the diagnosis as well as predict disease active status [5]. Soluble Interleukin-2 Receptor (IL-2R) level at the time of diagnosis may be a useful marker for prognosticating spontaneous remission or chronic disease course in sarcoidosis [6, 7]. Beijer et al. reported that serum amyloid A (SAA) levels were higher in fibrotic sarcoidosis patients compared to those without fibrosis [8]. Other investigators have reported that an increased neutrophil to lymphocyte ratio (NLR) can be seen in patients with lung parenchymal involvement and chronic sarcoidosis [9, 10]. However, the value of serum biomarkers has not been clearly identified for the prediction of multi-organ involvement or prognostic outcome assessment for patients with sarcoidosis. The aim of this study was to identify common biomarkers that could be used as tools to predict organ involvement and prognosticate outcomes in patients with sarcoidosis.

## Methods

### Data collection

Patients with newly diagnosed pulmonary sarcoidosis were enrolled in Shanghai Pulmonary Hospital between Mar 2013 and Sep 2021. Patient data were extracted and reviewed from electronic medical records. The diagnosis of sarcoidosis is based on the following criteria: a compatible clinical and imaging feature, histologic evidence of non-necrotizing epithelial granuloma, and the exclusion of alternative causes of granulomatous disease [3].

The 2014 revised WASOG instrument was used to determine the organ involvement of each patient [11]. The likelihood of specific organ involvement was categorized as highly probable, probable or possible according to prescribed clinical manifestation. When the biopsy was confirmed as non-necrotizing epithelial granuloma, or clinical manifestation meets either “highly probable” or “probable” disease category, the specific organ involvement was defined.

The prognostic outcome was defined as follows: Improved was considered when the clinical manifestations and chest High-Resolution Computed Tomography (HRCT) findings were considered significantly better during the follow up period. Persistent disease was defined as no change or disease worsening during the follow up period. The HRCT scan was independently reviewed by two thoracic radiologists who were blinded to clinical status and subject demographics. The protocol has been approved by the institutional review board of Shanghai Pulmonary Hospital (No. k21-390).

### Assessment of laboratory biomarkers

Patients underwent venous blood sampling for biomarkers during their initial visit for evaluation of sarcoidosis. According to the clinical practice guidelines for the diagnosis and detection of sarcoidosis [12], routine and important biomarkers analyzed included: white blood cell, neutrophil, lymphocyte and platelet counts, NLR, along with levels for hemoglobin, SAA, serum calcium, creatinine, alkaline phosphatase (ALP), SACE, IL-2R, Interleukin-6 (IL-6), tumor necrosis factor-α (TNF-α), serum ferritin (SF). Additionally, T-cell subsets were collected for cluster of differentiation 4+ (CD4+), CD8 + and the CD4 + T cell /CD8 + T cell ratio. Baseline values were recorded for all enrolled patients along with stratified biomarkers based on organ involvement.

### Statistics

Descriptive statistical data for each laboratory variable were listed, and the values of parameter variables were expressed as mean ± standard deviation (SD). Differences of variables between single organ involvement group and multiple organ involvement group were analyzed by unpaired Student’s t-test. According to Bonferroni’s correction method, P values of laboratory variables were corrected (Pc). Using the forward log rank (LR) method, a multivariate logistic regression model was performed to identify independent factors for multiple organ involvement. And then receiver operating characteristic (ROC) curve was plotted and area under curve (AUC) was calculated to clarify the diagnostic roles of the predicting factor. Statistical analysis was performed using SPSS 22.0 package software with P value of < 0.05 considered statistically significant.

## Results

### Clinical characteristics

**Fig. 1:**
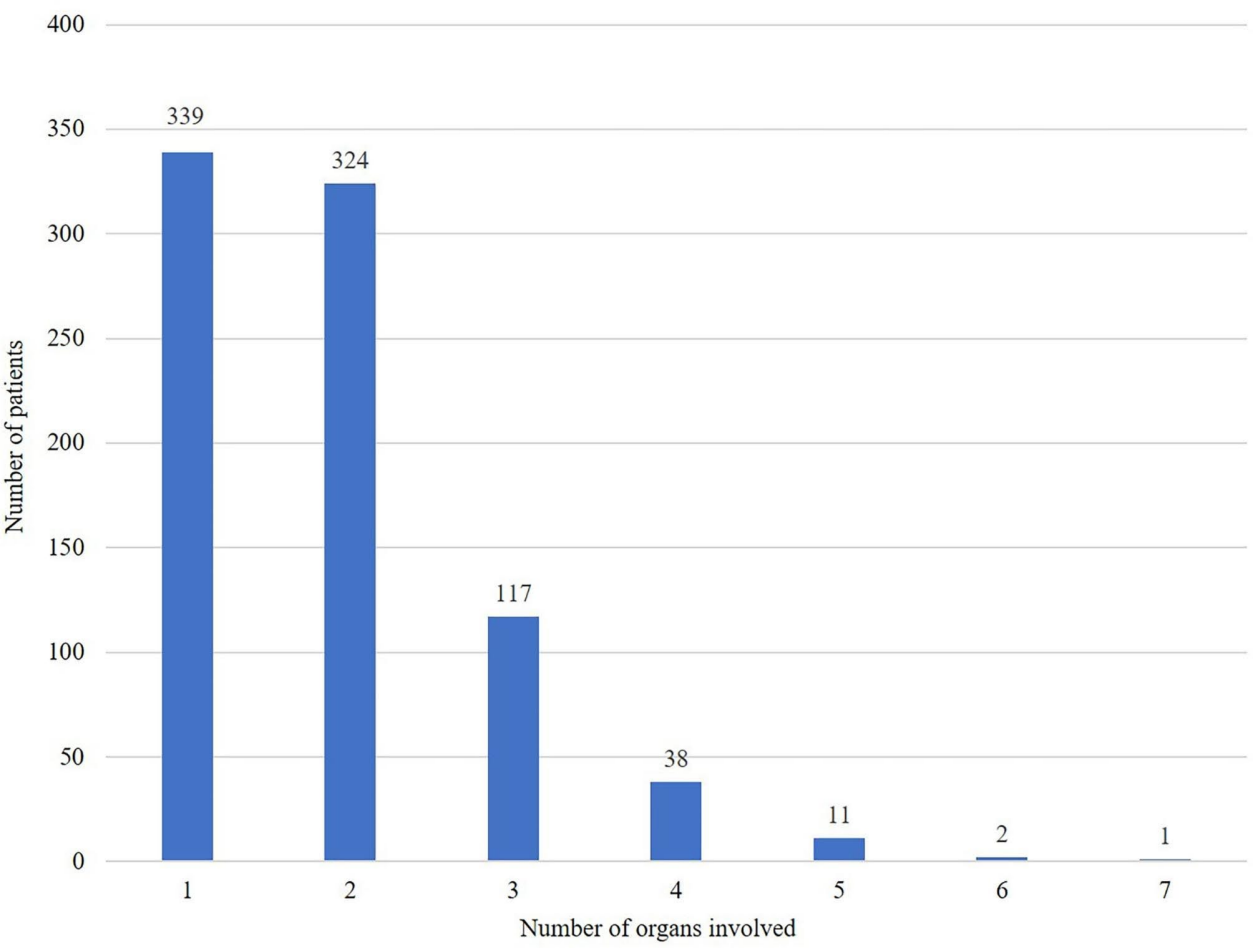
Distribution of the number of organs involved in 832 sarcoidosis patients

Table 1 lists the baseline clinical characteristics of the 832 patients with newly diagnosed pulmonary sarcoidosis from Mar 2013 to Sep 2021 in Shanghai Pulmonary Hospital. Patients included 550 (66.1%) females and 282 (33.9%) males, with a medium age of 52 years (females:54 years, males: 46 years, ranging from 20 to 76 years). Although the lung is the most common affected organ, a number of cases had multisystem involvement. A total of 350 (42.1%) cases had additional involvement in non-thoracic lymph node and 169 (20.3%) cases had abnormal calcium metabolism. There were 339 (40.7%) patients with single organ pulmonary involvement, and 493 (59.3%) patients with two to seven organs involved (Fig. 1). In the single organ involvement group, there were 254 (65.0%) females and 137 (35.0%) males, with a median age of 51 years old. In the multiple organ involvement group, there were 296 (67.1%) females and 145 (32.9%) males, with a median age of 53 years old. No significantly difference was noted in age and/or gender between one and multiple organs involvement groups.

**Table 1:** Characteristics of 832 Patients with Sarcoidosis

Characteristics	Number of patients	Percentages (%)
Gender (female)	550	66.1
Smoking history	103	12.4
Organ involvement		
Lung	830	99.7
Extra-thoracic lymph node	350	42.1
Calcium dysregulation	169	20.3
Skin	71	8.5
Spleen	49	5.9
Eye	36	4.3
Liver	18	2.2
Bone/joint	18	2.2
Parotid/salivary	9	1.1
Neurologic system	8	1.0
ENT	7	0.8
Heart	6	0.7
Renal	5	0.6
Muscle	5	0.6
Bone marrow	3	0.4
Scadding radiological staging		
0	4	0.5
I	216	26.0
II	545	65.5
III	27	3.2
IV	40	4.8

ENT: ear, nose and throat

### Routine serum biomarkers and organ involvement

Table 2 showed the laboratory biomarkers distribution in single and multiple organ involvement groups evaluated in this study. A total of 18 biomarkers were evaluated, of which two showed significant differences between the single organ and multiple organs affected groups. The SACE levels were significantly higher in patients with multiple organ involvement compared to those with single organ involvement (68.4 ± 39.6 vs. 53.8 ± 29.3, P/Pc < 0.001). Similarly, IL-2R levels were higher in the multiple organ involvement group (1220.8 ± 969.6 vs. 958.4 ± 695.2, P/Pc = 0.002/0.036). Although the neutrophil and platelet levels in the single organ involvement group were higher than those in the multiple organ involvement group, this was statistically insignificant after correction (3.8 ± 1.6 vs. 3.5 ± 1.4, P/Pc = 0.021/NS; 229.1 ± 63.5 vs. 218.7 ± 66.1, P/Pc = 0.022/NS, respectively). Table 3 showed a multivariate logistic regression model for one or multiple organ involvement. Only the SACE was an independent factor for multiple organ involvement (P = 0.018, hazard ratio (HR) = 1.009, 95% confidence intervals (95% CI) = 1.002–1.016).

**Table 2:** Biomarker Distribution in Single and Multiple Organ Involvement

Biomarker (No.)	Single Organ (means ± SD)	Multiple Organ (means ± SD)	*P/Pc* Value
WBC, ×10×/L (832)	5.8 ± 1.8	5.8 ± 6.2	NS
N, ×10×/L (832)	3.8 ± 1.6	3.5 ± 1.4	0.021/NS
L, ×10×/L (832)	1.4 ± 0.5	1.4 ± 1.5	NS
NLR (832)	3.0 ± 1.7	3.1 ± 1.8	NS
Hb, g/L (832)	133.1 ± 15.4	131.5 ± 13.7	NS
PLT, ×10×/L (832)	229.1 ± 63.5	218.7 ± 66.1	0.022/NS
SAA, mg/L (397)	14.2 ± 31.5	13.8 ± 26.1	NS
Serum Calcium, mmol/L (828)	2.3 ± 0.2	2.3 ± 0.2	NS
Creatinine, umol/L (832)	60.5 ± 15.0	59.8 ± 14.6	NS
AKP, U/L (788)	79.0 ± 29.8	80.1 ± 31.7	NS
SACE, U/L (784)	53.8 ± 29.3	68.4 ± 39.6	< 0.001/<0.001
IL-2R, U/ml (425)	958.4 ± 695.2	1220.8 ± 969.6	0.002/0.036
IL-6, pg/ml (466)	9.8 ± 22.0	13.7 ± 38.8	NS
TNF-α, pg/ml (437)	37.3 ± 67.5	41.2 ± 66.7	NS
SF, ng/ml (757)	102.0 ± 173.8	93.8 ± 75.7	NS
CD4 + T cell, % (627)	36.6 ± 10.3	36.4 ± 10.3	NS
CD8 + T cell, % (627)	18.4 ± 8.0	18.0 ± 8.3	NS
CD4 + T cell /CD8 + T cell (627)	2.5 ± 2.2	2.6 ± 1.6	NS

WBC: White blood cell; N: Neutrophil; L: Lymphocyte; NLR: Neutrophil to lymphocyte ratio; Hb: Hemoglobin; PLT: Platelet; SAA: Serum amyloid A; AKP: Alkaline phosphatase; SACE: Serum angiotensin converting enzyme; IL-2R: Interleukin-2 Receptor; IL-6: Interleukin-6; TNF-α:Tumor necrosis factor alpha; SF: serum ferritin; NS: No significant. SD: standard deviation

**Table 3:** Multivariate logistic regression model

Variables	P value	Hazard ratio	95% confidence intervals
N, ×10×/L	0.245	0.925	0.812–1.055
PLT, ×10×/L	0.065	0.997	0.994-1.000
SACE, U/L	0.018	1.009	1.002–1.016
IL-2R, U/ml	0.279	1.000	1.000–1.000

**Fig. 2:**
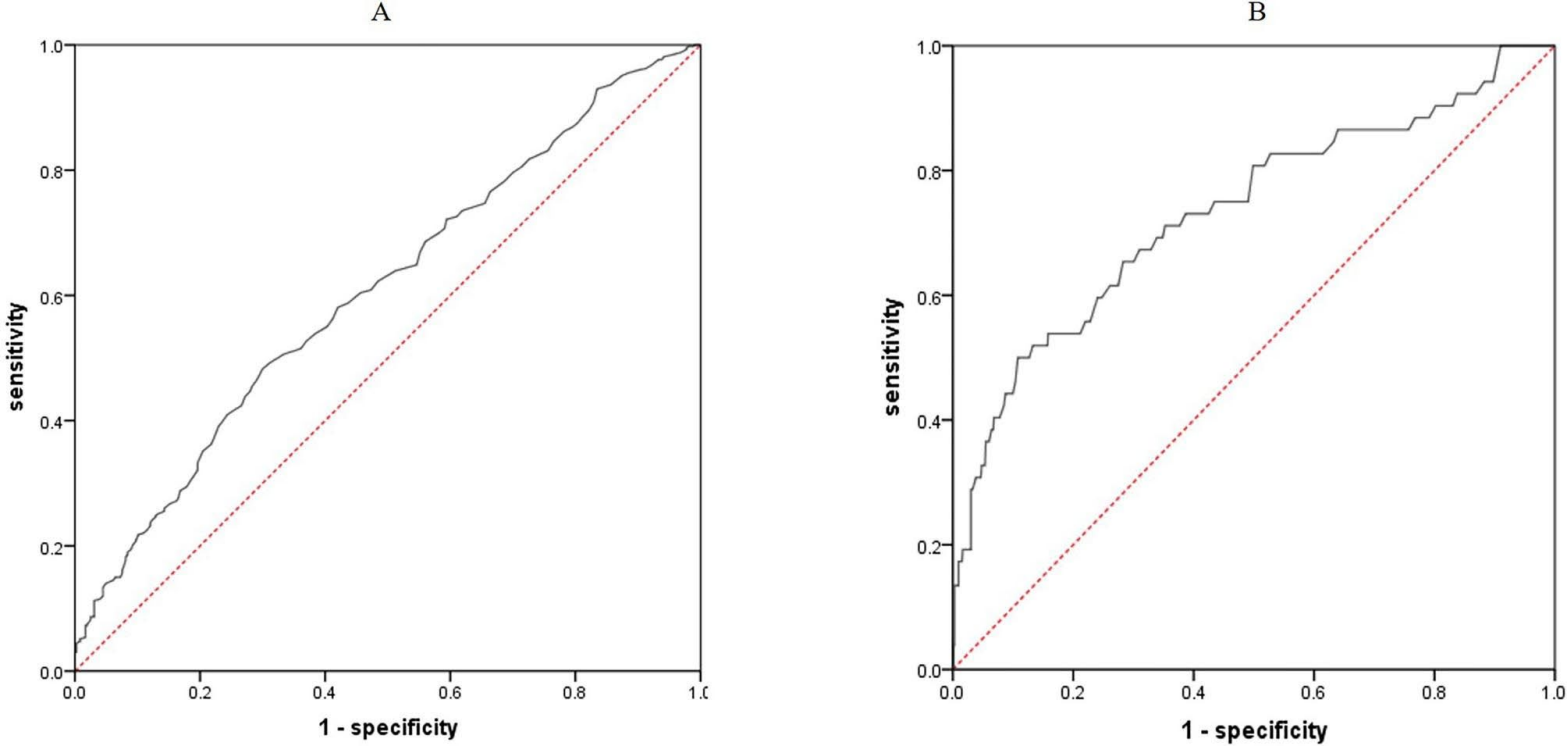
Diagnostic value for multiple organs involvement of SACE. (A) A cut-off SACE value of 61.5 U/L was obtained from ROC curve in single organ involvement and multiple organ involvement groups, and this cut-off value yielded a sensitivity of 48.2% and a specificity of 70.0%. The area under curve (AUC) value of the ROC curve is 0.608; and (B) A cut-off SACE value of 102.5U/L was obtained from ROC curve in three organ involvement and more than three organ involvement groups, and this cut-off value yielded a sensitivity of 50.0% and a specificity of 89.2%. The AUC value of the ROC curve is 0.734

ROC curve was plotted to evaluate the value of SACE in distinguishing sarcoidosis patients with single organ and multiple organ involvement. An optimal cut off SACE value of 61.5U/L was obtained, with the AUC value of 0.608. The sensitivity and specificity were 48.2% and 70.0% respectively (Fig. 2A). Comparing the two groups of patients with less than or equal to three organ involvement and more than three organ involvement. An optimal cut off SACE value was 102.5U/L, with the AUC value of 0.734. The sensitivity and specificity were 50.0% and 89.2% respectively (Fig. 2B).

**Fig. 3:**
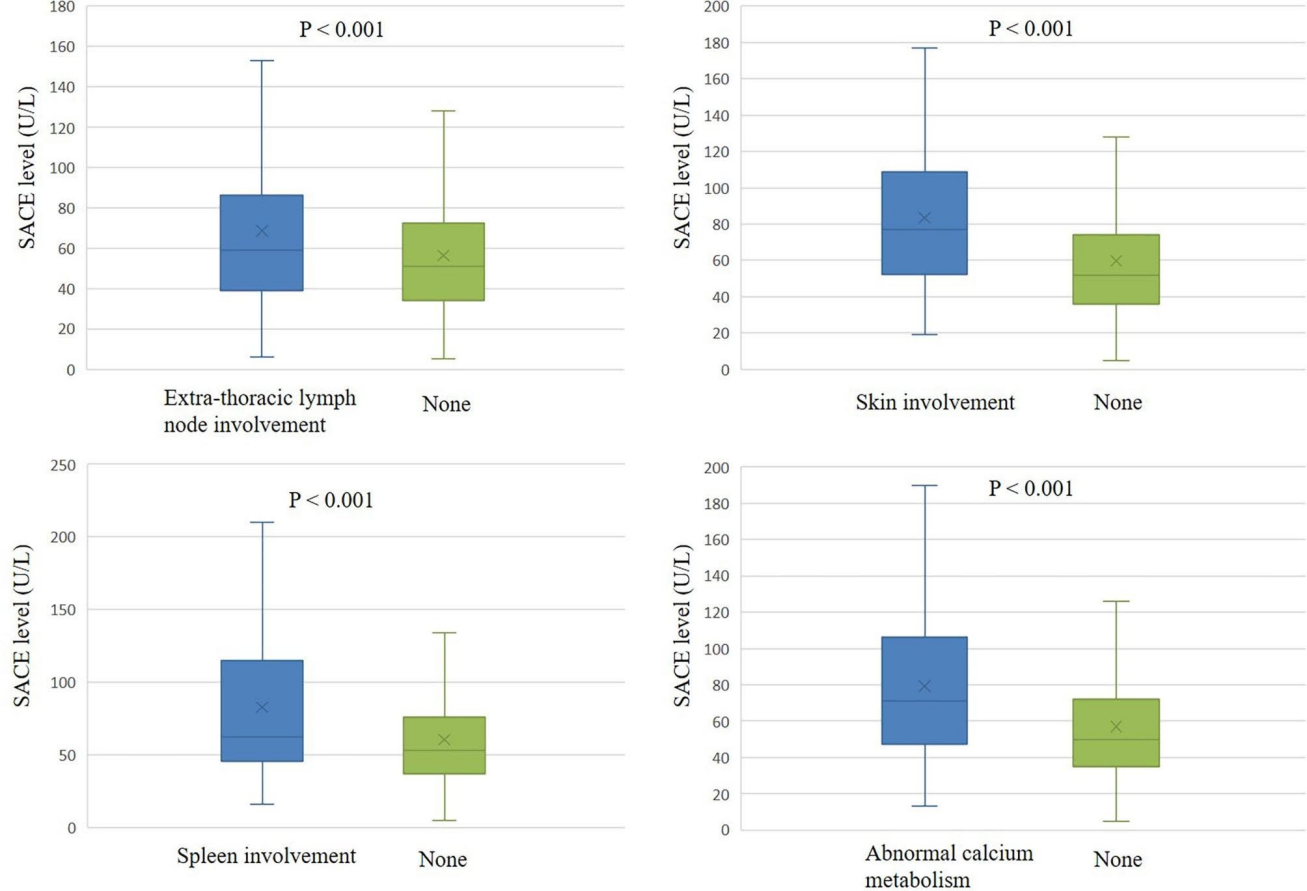
Evaluation of SACE levels in different organs involvement

**Fig. 4:**
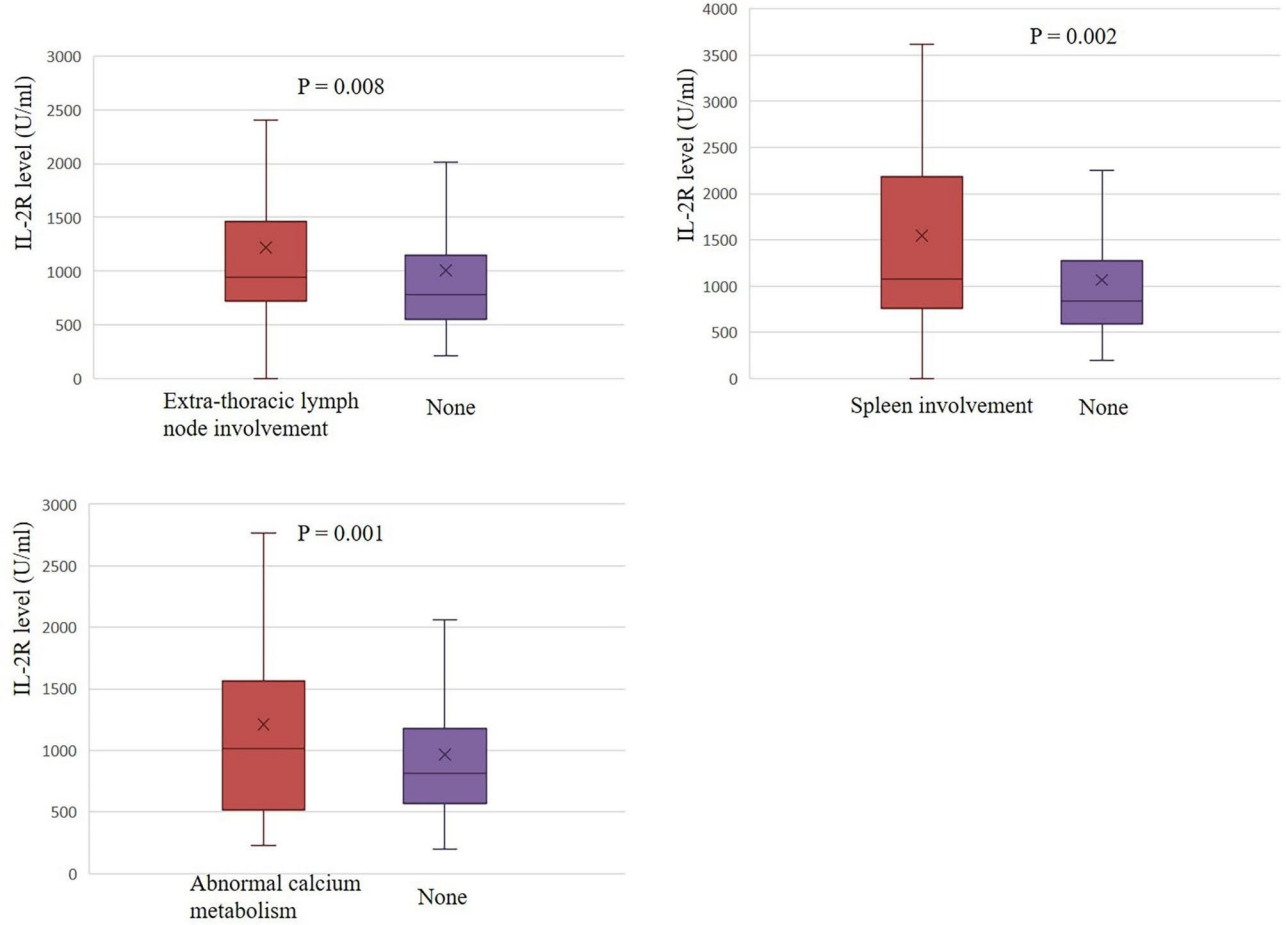
Evaluation of IL-2R in different organs involvement

**Fig. 5:**
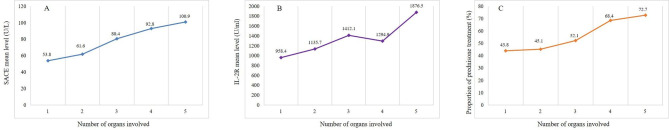
The levels of SACE and IL-2R, as well as the proportion of prednisone treatment all showed upward trend by the number of involved organs increased

SACE and IL-2R levels were evaluated for different organ involvement. SACE levels were higher in patients with extra-thoracic lymph node involvement (68.6 ± 41.1 vs. 56.5 ± 30.6, P < 0.001), skin involvement (83.4 ± 39.9 vs. 59.7 ± 35.0, P < 0.001), spleen involvement (86.2 ± 54.9 vs. 60.3 ± 34.0, P < 0.001) and abnormal calcium metabolism (86.3 ± 44.3 vs. 59.6 ± 34.4, P < 0.001) (Fig. 3). As shown in Fig. 4, IL-2R levels were higher in patients with extra-thoracic lymph node involvement (1229.8 ± 880.6 vs. 1004.3 ± 839.0, P = 0.008), spleen involvement (1620.6 ± 1195.0 vs. 1067.3 ± 829.2, P = 0.002) and abnormal calcium metabolism (1586.8 ± 1502.7 vs. 1058.8 ± 775.4, P = 0.001). In the levels of SACE or IL-2R between the groups with and without other organ involvement such as liver, eyes and bone/joint, there was no obvious difference. As the number of involved organs increased, the mean level of both SACE and IL-2R showed an upward trend (Fig. 5A and B).

### Treatment decision and prognostic evaluation

After diagnosis, 389 of 832 (46.8%) patients were prescribed glucocorticoids. The prescribed glucocorticoid usage significantly increased with multi-organ involvement, (P = 0.017). In the single organ involved group, the proportion of patients receiving treatment was 43.7% (146/339). As depicted in Fig. 5C, the higher the number of organs involved, the greater the proportion of patients received treatment. Compared with the non-treatment group, the levels of SACE in the treatment group were significantly increased (64.8 ± 39.7 vs. 58.8 ± 31.9, P = 0.019). Similarly, the levels of IL-2R in the treatment group were markedly higher than those in the non-treatment group (1271.2 ± 1034.2 vs. 947.4 ± 640.5, P<0.001).

**Fig. 6:**
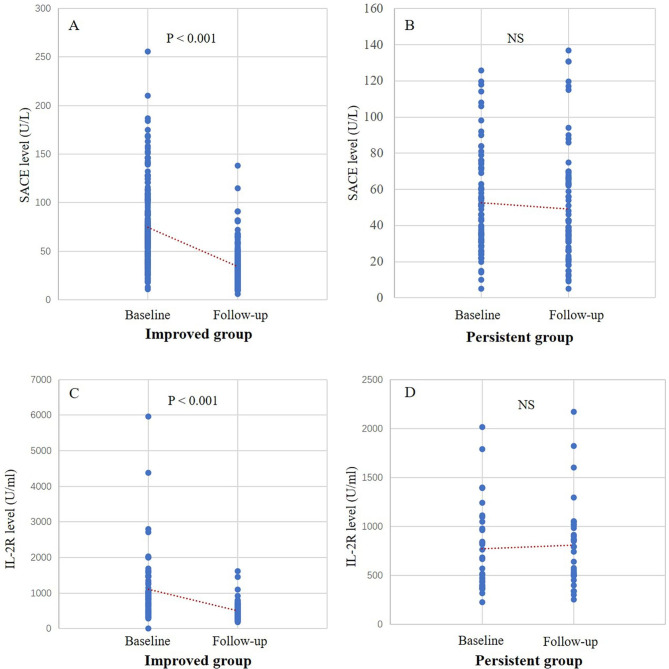
The levels of SACE and IL-2R on baseline and follow-up

There were 268 patients with follow-up SACE data and concurrent prognostic information (within one month). In this group, 191 patients improved and 77 patients experienced persistent disease. In the improved group, SACE levels were significantly decreased from 74.6 ± 41.0 to 34.5 ± 18.8 U/L(P<0.001)(Fig. 6A). In contrast, the SACE levels from the persistent patients remained unchanged from baseline to follow-up (Fig. 6B). As for IL-2R, there were 95 patients with both baseline and follow-up data. Improvement was noted in 64 patients, and other 31 patients experienced persistent disease. In the improved group, IL-2R levels were significantly decreased from 1099.6 ± 935.8 to 501.3 ± 261.6 U/ml (P<0.001)(Fig. 6C). However, in the persistent group, the IL-2R follow-up levels were unchanged from baseline (Fig. 6D).

## Discussion

This study investigated the utility of serum biomarkers on predicting organ involvement and prognostic monitoring in patients with sarcoidosis. In this study cohort, there were 40.7% of patients with single organ involvement, and 59.3% of patients witnessed two to seven organs involved. Among the 18 potential serum biomarkers evaluated in this study, only SACE and IL-2R levels were obviously higher in multiple organs involved cases than those with single organ involvement. In the multivariate logistic regression models analysis, only the degree of SACE was an independent factor of multiple organs involvement. SACE levels increased in patients with extra-thoracic lymph node, skin, spleen involvement along with abnormal calcium metabolism. IL-2R levels generally increased in cases with the multi-organ involvement of extra-thoracic lymph node, spleen and abnormal calcium metabolism. From a prognostic aspect, in the improved group, both SACE and IL-2R levels decreased significantly from baseline. In contrast, no change in biomarker levels was found in those patients with persistent disease. These findings suggest that the evaluation of biomarkers SACE and IL-2R at sarcoidosis diagnosis may help to predict multi-organ involvement and prognosticate sarcoidosis outcome.

SACE is mainly produced by the epithelioid cell in the sarcoidosis granuloma that converts angiotensin I into angiotensin II [13]. Due to insufficient sensitivity and specificity as a diagnostic tool, test of SACE had been considered more meaningful in evaluating the activity of sarcoidosis granulomas than in determining the diagnosis [14]. In a meta-analysis, the sensitivity and specificity of SACE was reported as 0.76 and 0.80 respectively for estimating the active status of sarcoidosis [5]. Research on the correlation between biomarkers and organ involvement has been studied recently. Wang et al. observed that SACE values were higher in systemic involved sarcoidosis cases compared to those with only intrathoracic sarcoidosis, with the mean SACE level of 64.94 ± 39.72 U/L and 47.05 ± 23.66 U/L respectively (P = 0.009) [15]. In another retrospective study, Yasar et al. reported that SACE values were higher in extra-thoracic involvement group than in those without it [16], and ROC curve analysis revealed an AUC of 0.816, 70.6% sensitivity and 80% specificity at the SACE cut-off value of 197.5 U/L. In a study of Czech patients, the SACE and IL-2R levels were insignificantly higher in those with pulmonary plus extra pulmonary disease versus pulmonary alone [17]. Our findings from Chinese Han population support the correlation between SACE and multiple organ involvement. The cut off SACE of 61.5U/L was obtained for distinguishing sarcoidosis patients with single organ and multiple organ involvement, which was similar to the previous study in China with the mean SACE level of 64.94 U/L in systemic involved sarcoidosis cases [15]. However, there was obvious difference in cut off SACE value between our study and Yasar’ study in Turkey (61.5U/L and 197.5 U/L respectively), which may due to differences in race and geography. Furthermore, this study suggests an upward trend in SACE levels as the number of involved organs increases. In addition, we revealed the role of SACE in specific organ involvement: extra-thoracic lymph node, skin, spleen and abnormal calcium metabolism.

IL-2R was expressed on the cell surface by the activation of T helper cells in granulomatous inflammation sites. Subsequently, the soluble IL-2R molecules fall off and enter the microcirculation. IL-2R reflects the activity of the T-helper cell component and may play a role to assess disease activity and prognosticate [6, 18]. Reithmann et al. suggested the IL-2R was superior to SACE in detecting the inflammatory activity of cardiac sarcoidosis patients with ventricular arrhythmias [19].

Other investigators found IL-2R was a good biomarker to identify multiple organs involvement or extrapulmonary disease in sarcoidosis patients [18, 20]. Consistent with these previous small sample research, our study confirmed the serum IL-2R level in multiple organ involvement group was significantly higher than that in single organ involvement. and showed IL-2R level was correlated well with the number of organs involved for sarcoidosis patients. There were few studies about biomarkers and specific organ involvement. In this study, correlation between IL-2 level and extra-thoracic lymph node, spleen involvement and abnormal calcium metabolism had been revealed.

SACE and IL-2R may be useful in disease monitoring. The present study demonstrates that SACE levels and IL-2R levels had a significantly decline between baseline and follow-up in an improved group, while there was no significant difference in levels from a persistently active group, regardless of treatment status. Reports suggest that with spontaneous disease resolution or therapeutic control in patients receiving corticosteroids or immunosuppression elevated SACE levels normalize [21, 22]. The fall of SACE with therapy may be due to resolution of disease or direct suppression of SACE by corticosteroids [23]. Schimmelpennink et al. suggested high levels of sIL-2R might serve as a prognostic biomarker for chronic sarcoidosis [7]. The positive correlation between the decline of SACE or IL-2R levels and the state of disease remission has been revealed in this study, strengthening the viewpoint that these two biomarkers may reflect the granuloma burden or activity in sarcoidosis.

There were several limitations in this study. First, this study was retrospectively conducted in a single pulmonary hospital and may be not representative of other sarcoidosis cohorts. Virtually all patients were diagnosed with pulmonary sarcoidosis. Hence the findings may not extrapolate to other single sites of granulomatous sarcoid inflammation such as eye, heart, or nervous system. Second, the follow-up population size was relatively small. Finally, we did not examine the polymorphism of ACE or IL-2R in the patients. Therefore, prospective and multicenter studies are necessary to obtain results with higher evidence-based grade.

## Conclusion

The biomarkers SACE and IL-2R were found useful as routine serum biomarkers in the initial evaluation of organ involvement as well as monitoring prognosis in sarcoidosis. In patients with sarcoidosis, SACE and IL-2R levels decreased more in patients with disease improvement compared to those with persistent disease.

## Data Availability

The datasets used during the current study are available from the corresponding author on reasonable request.
